# In Vitro Evaluation of Ozonated Water Treatment on the Viability of *Eimeria* Oocysts and *Giardia* Cysts from Water Buffaloes: A Proof-of-Concept Study

**DOI:** 10.3390/vetsci8060115

**Published:** 2021-06-18

**Authors:** Maria Elena Morgoglione, Antonio Bosco, Lavinia Ciuca, Paola Pepe, Gerald C. Coles, Giuseppe Cringoli, Laura Rinaldi

**Affiliations:** 1 Department of Veterinary Medicine and Animal Production, University of Federico II, Via Delpino, no. 1, 80137 Napoli, Italy; elenamorgoglione@gmail.com (M.E.M.); boscoant@tiscali.it (A.B.); paolapep04@yahoo.it (P.P.); cringoli@unina.it (G.C.); lrinaldi@unina.it (L.R.); 2 Ubley Biologics, P.O. Box 170, Ubley, Bristol BS40 6JA, UK; gerald.coles@cantab.net

**Keywords:** protozoa, buffaloes, ozone, control, alternative strategies

## Abstract

The aim of this proof-of-concept study was to evaluate the in vitro effects of ozonated water treatment on the viability of *Eimeria* oocysts and *Giardia* cysts isolated from naturally infected water buffaloes. *Eimeria* oocysts were divided into seven groups of six replicates that were treated with ozonated water at three ozone concentrations (0.5, 1, and 2 mg/L) and two contact times (five and ten minutes), and one group (negative control) that was exposed to non-treated water. *Giardia* cysts were divided into nine groups of six replicates and were treated with ozonated water at four ozone concentrations (0.1, 0.3, 0.5, and 1 mg/L) and two contact times (one and two minutes), while one group (negative control) was exposed to non-treated water. The results of the ozonated water treatment gave a 33% inhibition of the sporulation of *Eimeria* oocysts and rendered 96.3% of *Giardia* cysts non-viable, suggesting that ozonated water treatment could be a promising alternative sanitation technology to common conventional disinfectants for reducing intestinal protozoa infections in water buffaloes; though further in vitro and in vivo tests are needed.

## 1. Introduction

Dairy water buffalo (*Bubalus bubalis*) farming plays an important role in the economy of several countries, including Italy, as their milk is almost exclusively used for the production of-mozzarella-cheese [1,2]. In intensive farming systems, the infection of water buffaloes with intestinal protozoa, such as *Giardia* and *Eimeria*, threatens the profitability and sustainability of milk production [3,4,5]. These parasites are the leading cause of neonatal diarrhea, with negative impacts on the growth performance of buffalo calves, resulting in economic losses [6,7]. Water buffaloes acquire *Eimeria* infections soon after birth, and severe outbreaks can occur, resulting in morbidity and mortality regardless of the management system (intensive, semi-intensive, or extensive) of the water buffalo farm. Although metaphylactic approaches have been used successfully to control *Eimeria* (e.g., toltrazuril and diclazuril) and *Giardia* (e.g., fenbendazole and albendazole) infections in ruminant farms, other prophylactic measures are needed to reduce environmental contamination, in order to limit the infection pressure [8,9]. Moreover, infection by *Giardia duodenalis* is a public health concern, because of the potential zoonotic transmission to humans [10]. Therefore, the adoption of appropriate control strategies against intestinal protozoa is a considerable challenge for water buffalo farms [11]. Water plays an important role in a wide range of operations, including cleaning and disinfection, that are carried out in the dairy industry [11,12,13]. Dairy wastewaters are conventionally purified by physicochemical and biological methods [13]. However, a number of studies have been published on the use of ozone treatment, either alone or in combination with other technologies, in order to reuse, at least in part, the wastewater produced by the dairy sector [14,15,16,17].

Ozone, an allotropic form of oxygen constituted by three oxygen atoms, is a very powerful oxidant, and it is well known for its bactericidal, viricidal, and fungicidal actions, which are used for water treatment and medical applications [18,19,20,21,22]. Ozone has been reviewed by the European Environmental Agency for use as a biocide, in disinfection, food and animal feeds, drinking water, and as a preservative for liquid systems, under the Biocidal Products Regulation (BPR) of the European Chemical Agency (ECHA). In Italy, ozone can be used exclusively as a sanitizer.

In the last few years, the scientific and commercial interest in ozone-therapy, both in human and veterinary medicine has been increasing [22,23]. The main advantage of using ozonated water treatment in the livestock and animal husbandry sectors is the ability for reducing or destroying microbial pathogens, thus resulting in an improvement of the general health of the animals [24,25,26]. 

Given the potential applications in veterinary medicine, the aim of this proof of concept study was to evaluate the in vitro effect of ozonated water on *Eimeria* oocysts and *Giardia* cysts isolated from naturally infected water buffaloes.

## 2. Materials and Methods

### 2.1. Sampling and Coprological Analysis

**Figure 1:**
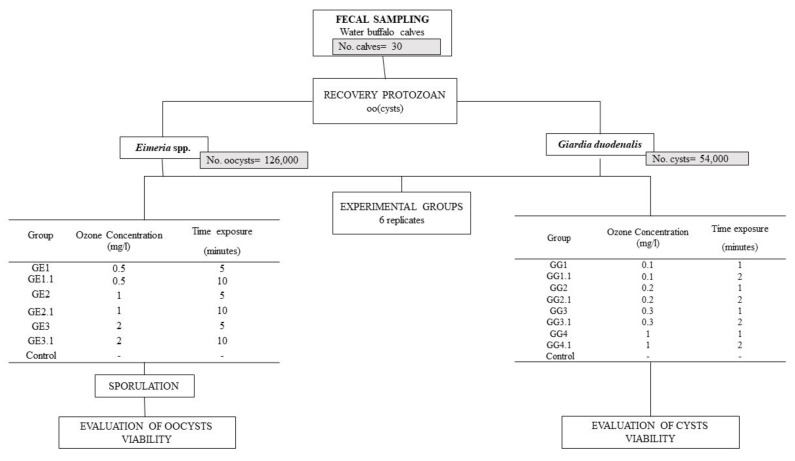
Study design of the in vitro effect of ozonated water treatment on the viability of *Eimeria* spp. oocysts and *Giardia duodenalis* cysts isolated from naturally infected water buffaloes.

*Eimeria* oocysts and *Giardia* cysts were recovered from fecal samples collected from naturally infected water buffalo calves (1–4 months), on a farm located in southern Italy with a known history of protozoa infections. Thirty individual fresh fecal samples were collected directly from the rectal ampulla of the animals. Each fecal sample was analyzed by the FLOTAC dual technique with an analytic sensitivity of two oocyst/cysts per gram (OPG and CPG, respectively) of feces, using two flotation solutions, namely sodium chloride (specific gravity, s.g. = 1.20) to detect *Eimeria* oocysts [27] and zinc sulfate (s.g. = 1.35) to detect *G. duodenalis* cysts [28]. Magnifications of 100× and 400× were used to identify protozoan (oo)cysts. The positive samples with a mean value of 30,000 OPG for *Eimeria* and with a mean value of 15,000 CPG for *G. duodenalis* were processed to purify the (oo)cysts for the in vitro tests. The tests were organized as described in Figure 1 and in the following sections.

### 2.2. Recovery of Eimeria spp. Oocysts and Giardia Duodenalis Cysts 

The fecal samples were processed within two hours of collection using the egg recovery technique of Bosco et al. [29] with some modifications. Briefly, the fecal samples were homogenized and filtered under running water through sieves with different mesh sizes, as follows: 1 mm, 250 μm, 100 μm, and 50 µm, to separate the (oo)cysts from the feces; with a sieve of 25 μm for *G. duodenalis*. The fecal suspension obtained was centrifuged for three minutes at 170 g, and the supernatant was discarded. Finally, the pellets were resuspended with 40% sucrose solution and transferred to new tubes. To obtain a clear aqueous solution with *Eimeria* spp. oocysts and *Giardia* cysts, two successive rounds of centrifugation with distilled water were performed. After a thorough homogenization (avoiding foam formation) of the suspension into the tubes, ten aliquots of 10 μL each were taken in order to count the number of *Eimeria* oocysts or *Giardia* cysts at 100× and 400× magnifications.

### 2.3. Water Ozonisation

The ozonated water was generated in a small-scale circuit by passing the distilled water through an electrolytic cell with a current of 1A (amps). The water was continuously pumped past the electrode at rate of 1 L/min to produce ozone [30]. A DPD (N, N-diethyl-p-phenylenediamine) colorimetric glycine method for residual chlorine using a compact ozone meter (Palintest^©,^ Camlab Ltd., Cambridge, UK) was employed to measure the ozone concentration [31,32,33]. The output of a preliminary experiment revealed that the concentration of aqueous ozone varied with temperature. The treated solution showed near-saturation with ozone after one minute of treatment. However, the ozone treatment in subsequent tests was set at an average temperature of 25 °C with pH = 7 [34,35,36]. In order to evaluate the sanitation kinetics, the concentration–time concept was applied, i.e., ozone concentration (C) in mg/L was multiplied by contact-time (*t*) in minutes (C*t*) [34,35].

### 2.4. In Vitro Test for Eimeria spp.

*Eimeria* oocysts (total count = 126,000) were randomly divided into seven groups (Group *Eimeria*-GE) of six replicates each in 42 glass vials, as follows: six groups (GE1; GE1.1; GE2; GE2.1; GE3; GE3.1) were treated with ozonated water at three ozone concentrations (0.5, 1, and 2 mg/L) and two contact times (5 and 10 min). One group was exposed to non-treated water (negative control), as shown in Table 1. The aliquots of all the groups were centrifuged for 3 min at 170 g and the pellet was resuspended in an aqueous solution of potassium dichromate 2.5% and incubated in a wide-surfaced container at 26–28 °C for 60–72 h, oxygenating the samples several times a day to preserve the oocysts [36]. At the end of the incubation, each aliquot was centrifuged for three minutes at 170 g, the supernatant was discharged to remove potassium dichromate from the oocyst suspension by repeated dilution with distilled water. Then, the aliquots were stored at 4 °C until being counted. Each aliquot was examined by light microscopy at 100×, 400×, and 1000× magnifications. In order to evaluate the effect of ozonated water treatment, the number of sporulated and non-sporulated, deformed, or lysed oocysts were counted, and the percentage of sporulation was estimated by counting the number of sporulated oocysts out of a total of 100 oocysts [36]. 

### 2.5. In Vitro Test for Giardia Duodenalis

*Giardia duodenalis* cysts (total count = 54,000) were divided into nine groups (Group *Giardia*-GG) of 6 replicates each (54 glass vials) as follows: eight groups (GG1; GG1.1; GG2; GG2.1; GG3; GG3.1) were treated with ozonated water at four ozone concentrations (0.1, 0.3, 0.5, and 1 mgLl) and two contact times (one and two minutes) (Table 2). One group was exposed to non-treated water (negative control). *Giardia* cyst viability was evaluated by non-fluorogenic dye exclusion method with trypan blue [37,38,39]. The percentage of non-viable cysts was calculated using the formula: (1 − (total number of viable cysts per ml of aliquot /total number of cysts per ml of aliquot)) × 100.

### 2.6. Statistical Analysis

One-way ANOVA was performed to detect the significant difference between the treated and non-treated groups of GE and GG with post hoc Turkey’s tests. For all comparisons, a level of *α* = 0.05 was assumed, and the obtained *p*-values were rounded to two decimal places. Statistical analysis were performed using SPSS Statistics v.23 (IBM, Armonk, NY, USA).

## 3. Results

### 3.1. Eimeria Oocysts Viability

**Figure 2:**
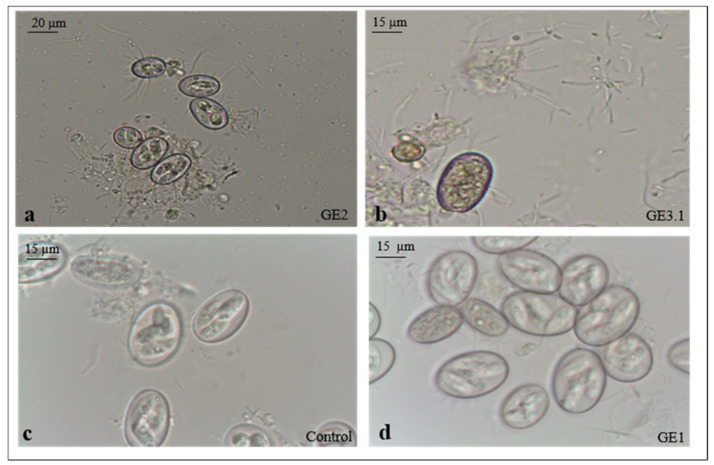
Presence of bacteria in the GE2 and GE3.1 treated groups (**a**,**b**) and in the control group (**c**); oocysts at early cytoplasmatic stage (**d**).

Based on sporulation, four species of *Eimeria* were identified in the positive samples, i.e., *E. ellipsoidalis, E. bovis*, *E. subspherica* and *E. bareillyi,* with the following prevalence: 42%, 29%, 18%, and 11%, respectively. The sporulation rate of the oocysts treated with ozone was significantly (*p* < 0.001) lower than that in the non-treated group (Table 3). The best results were obtained in the GE3 treated group with a *Ct* value of ten (2 mg/L * 5 min), which revealed a rate of 33.0% of non-sporulated oocysts. There was no significant difference (*p* > 0.005) between the prevalence of the four *Eimeria* species identified in both the treated and non-treated groups. Moreover, after incubation, a fast attachment of the bacteria to the surface of the ozone-treated oocysts was observed, unlike the non-treated control oocysts, where the attachment was minimal and delayed (Figure 2a–c). However, the oocysts showed internal structure degeneration, and the sporulation process was incomplete (Figure 2d).

### 3.2. Giardia Duodenalis Cysts Viability

**Figure 3:**
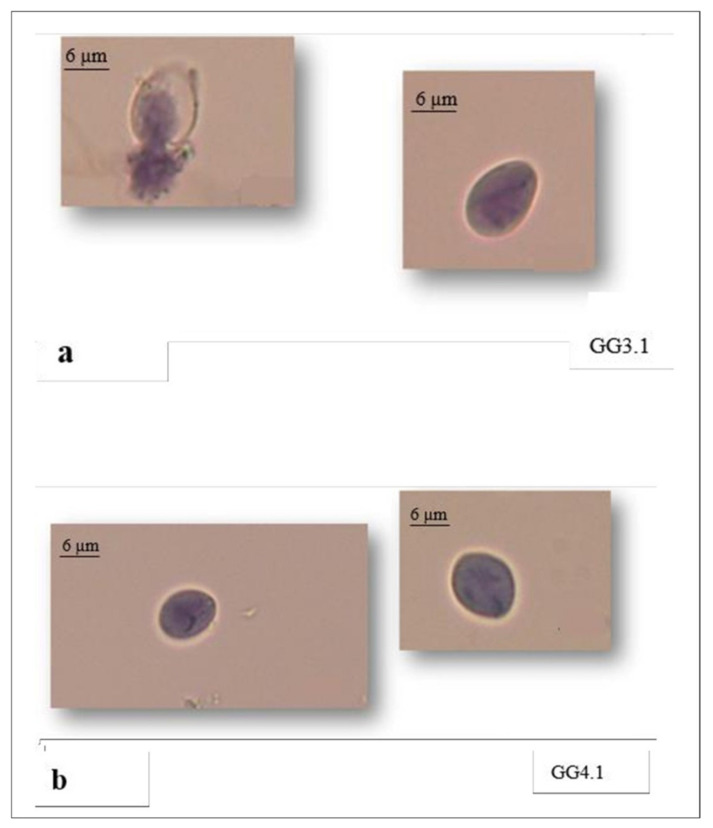
Non-viable cysts of *Giardia* in GG3.1 (**a**) and GG4.1 (**b**) treated groups.

The control group showed 99% intact *Giardia* cysts. There was a significant difference (*p* < 0.0001) between the mean viable cysts and the mean of non-viable cysts in the GG.1 treated group and non-treated group. Specifically, the highest percentage of non-viable cysts was obtained in the GG3.1 treated group (96.3%), at *Ct* of 1 (0.5 mg/L * 2 min) (Table 4) (Figure 3a,b).

## 4. Discussion

Ozone is well known for its strong oxidizing ability, and its action against protozoa has been proved in vitro with several parasites, such as *Leishmania*, *Giardia*, *Cryptosporidium*, *Blastocystis*, *Cyclospora*, etc., using ozonated oil and ozonated water [40,41,42,43,44]. In addition, ozone is being widely used in drinking water and wastewater treatment [45,46,47,48,49,50] as well as in human and veterinary medicine in several pharmaceutical forms [20,25,50,51,52,53,54]. This proof of concept study was the first attempt to evaluate the in vitro effectiveness of ozonated water treatment on the viability of *Eimeria* spp. oocysts and *G. duodenalis* cysts isolated from water buffaloes. 

Our study revealed that the inhibition of the sporulation of the *Eimeria* oocysts induced by the ozone treatment was time and concentration dependent. Indeed, the *Eimeria* oocysts suffered a partial inhibition of sporulation in the treated group (GE3) with 2 mg/L ozone concentration and 5 min of exposure. This might have been due to the alteration of the surface structure of the oocysts by ozone, as has been shown in other similar studies for *E. colchici*, *E. necatrix*, *E. maxima*, and *E. acervuline* oocysts [36,55]. Coccidian oocysts are extremely resistant to common physical and chemical compounds due to their complex structure [36,56,57,58]. However, in the present study, many oocysts after ozone treatment showed a deformed shape, incomplete development, and remained at the early cytoplasmic contraction stage. It should be noted, however, that the study by Liou et al. [36] reported ozone-treated *E. colchici* oocysts as being infective after 3 months, even if their sporulation was incomplete. Therefore, our results are not conclusive regarding infectivity, and further studies should be carried out for the buffalo *Eimeria* species identified in the present study. Ozone alone or in combination with other chemical (chlorine, chlorine dioxide) or physical processes (e.g., filtration, flocculation, UV, etc.) has been reported to inactivate the cysts of *Giardia* [45,52,59].

In the present study, the highest percentage of non-viable cysts was obtained in the GG3.1 treated group at 0.3 mg/L ozone concentration and 2 min time exposure (96.3%). Our results were similar to those obtained by Finch et al. [52] where the highest percentage of *G. duodenalis* inactivation cysts (99.9%) was obtained with a 0.5 mg/L ozone concentration and 5 min time exposure.

*Eimeria* spp. and *G. duodenalis* are still common and widespread GI parasites in water buffalo farms in different parts of the world [4,5,6,59,60]. In the Mediterranean area, *Eimeria* spp. is still the most prevalent protozoa, with an overall rate of 81.5%, according to Morgoglione et al. [61]; whilst *G. duodenalis* was present in buffalo farms in central and southern Italy, with a rate of 30% and 18%, respectively [9,61]. *Giardia* is present in buffalo farms with a wide range of prevalence, from 0.7% to 40.9% worldwide [5]. Furthermore, molecular investigations of *G. duodenalis* isolates revealed the presence of zoonotic parasites (*G. duodenalis* assemblage A) and host-specific parasites (*G. duodenalis* assemblage E), indicating that water buffaloes can have a major impact on environmental contamination, and with cysts potentially infectious to humans if their feces are not properly disposed of [10]. 

Recent studies have shown that water is one of the main sources of infection with *Cryptosporidium* spp. and *G. duodenalis,* and these (oo)cysts are unlikely to be inactivated by routine chemical disinfectants or sanitizing water treatments [62,63]. Thus, drinking water sanitation plays an important role in the complex control of protozoa in water buffalo farms, reducing environmental contamination pressure and protecting animals from infections. Prevention of overcrowding, feeding off the ground, and sanitation of feeding and watering equipment are important. Hence, these approaches represent a relevant challenge in intensive livestock farming [48]. Moreover, the control of intestinal protozoa in buffalo farms is certainly not easy to implement because of the considerable spread and resistance of their (oo)cysts in the environment and in water for months [7,49]. To date, as vaccine prophylaxis measures to control of GI protozoa are not yet available in ruminants, the best combination of rational treatments, hygiene, and herd management are indispensable tools to reduce the risk of transmission of infections in water buffalo farms [56]. 

In recent years, the scientific community has had an increased interest in new low-cost and eco-friendly systems to control parasites, thus alternative therapeutic approaches based on ozone in ruminants could be useful for controlling protozoa infections, as demonstrated in poultry against *Eimeria* [21,36]. Although the use of ozone in veterinary medicine can be traced back more than 30 years, it is still rarely employed, and only for the treatments of a few diseases, such as mastitis, vaginitis, enteritis, etc., to reduce antibiotic administration [21,22]. Furthermore, ozone has been successfully used in the dairy industry for cleaning operations in milk processing and for reducing the concentrations of pollutants in dairy wastewaters [51]. Despite the advantages of ozone, some limitations are associated with the ozonated water technology, such as the high cost of ozone generators, the need for operating and service infrastructure on a large scale, which could explain the limited use of ozone in livestock farms. With electrodes, such as those used in this study, and with the development of new boron doped diamond electrodes, it is now possible to generate ozone from water at the point of use. The cells required for this purpose provide a simple, robust, reliable, low voltage, and low cost method [64]. 

The Ct value is actually a measure of disinfection effectiveness for the time that the water and disinfectant are in contact, where “C” is the disinfectant residual concentration (measured in mg/L) at peak hourly flow, and “t” is the time (measured in minutes) that the disinfectant is in contact with the water at peak hourly flow. The contact time (t) is measured from the point of disinfectant injection to a point where the residual concentration is measured. Nevertheless, Ct values are widely used to calculate the dosage of disinfectants in water treatment, and they are particularly relevant for chlorine and ozone [65]. Moreover, the Environmental Protection Agency (EPA) provides specific Ct values for the inactivation of the *Giardia* cysts by ozone at pH 6–9. In our study, the Ct values for ozone to inactivate the *Giardia* cysts were similar to those reported by the EPA. However, there are no data regarding the Ct values for ozone to inactivate the *Eimeria* oocysts, therefore, our study highlighted preliminary findings on the Ct values of ozonated water treatment for the inhibition of the sporulation of *Eimeria* oocysts.

## 5. Conclusions

The results achieved represent the “first attempts” in evaluating the applicability of ozone as a water sanitizer in water buffalo farms. However, for a complete understanding of the mechanisms of action of ozone as a therapeutic agent on the infectivity of *Eimeria* spp. and *G. duodenalis*, it would be useful to perform in vitro tests to evaluate the efficacy of ozone against reproductive forms cultivable in vitro, e.g., sporozoites of *Eimeria* and trophozoites of *Giardia.* A hypothetical higher sensitivity of these forms to the treatment with ozone in vitro would allow, after the experimental infection of animals with oo/cystic forms (i.e., in vivo), highlighting the ability of ozone to reduce the excretion (as well as the diffusion) of parasitic oo/cysts in the environment, while providing data on the “prophylactic” capacity of ozonated water. No data are available on the effect of ozone on the infectious stages of *Eimeria* spp. However, the outcomes of our proof-of-concept study suggest ozonated water treatment is a promising alternative to the commonly used disinfectants for reducing intestinal protozoa infections in water buffaloes; though further in vitro and in vivo tests are needed. 

## Figures and Tables

**Table 1:** In vitro tests for *Eimeria* oocysts using different ozone concentrations and time exposures (minutes) in the GE.

Group-*Eimeria* (6 Replicates)	Ozone-Concentration (mg/L)	Time Exposure (Minutes)	*Ct* * Value
GE1	0.5	5	2.5
GE1.1	0.5	10	5
GE2	1	5	5
GE2.1	1	10	10
GE3	2	5	10
GE3.1	2	10	20
Control	-	-	-

* *Ct* is expressed by ozone concentration (C) in mg/L multiplied by contact-time (t) in minutes.

**Table 2:** In vitro tests for *Giardia* oocysts using different ozone concentrations and time exposure (minutes) in the GG.

Group-*Giardia* (6 Replicates)	Ozone-Concentration (mg/L)	Time Exposure (Minutes)	*Ct* * Value
GG1	0.1	1	0.1
GG1.1	0.1	2	0.2
GG2	0.2	1	0.2
GG2.1	0.2	2	0.4
GG3	0.3	1	0.3
GG3.1	0.3	2	0.6
GG4	1	1	1
GG4.1	1	2	2
Control	-	-	-

* *Ct* is expressed by ozone concentration (C) in mg/L multiplied for contact-time (t) in minutes.

**Table 3:** The sporulation rate of the *Eimeria* spp. oocysts treated with ozonated water.

Group-*Eimeria* (6 Replicates)	Viable Oocysts (Sporulated) (%)
GE1	83.2
GE1.1	82.6
GE2	77.2
GE2.1	76.5
GE3	77.0
GE3.1	76.3
Control	89.0

**Table 4:** Effect of ozone on the *Giardia duodenalis* cyst viability.

Group-*Giardia* (6 Replicates)	Non-Viable Cysts (%)
GG1	16.2
GG1.1	34.5
GG2	86.3
GG2.1	94.0
GG3	76.8
GG3.1	96.3
GG4	90.8
GG4.1	95.2
Control	2.0

## Data Availability

Data is contained within the article.
